# Successful Treatment of Refractory Anemia in a Patient With Glycogen Storage Disease Type Ia Undergoing Hemodialysis

**DOI:** 10.7759/cureus.26213

**Published:** 2022-06-22

**Authors:** Hirotaka Sato, Kentaro Takase, Seikon Kin

**Affiliations:** 1 Nephrology, Shimane Prefectural Central Hospital, Izumo, JPN

**Keywords:** iron deficiency anemia (ida), anemia of chronic disease (acd), hepatic adenoma, hepcidin, glycogen storage disease type 1a, maintenance hemodialysis

## Abstract

Glycogen storage disease type Ⅰa (GSDIa), also known as von Gierke disease, is a rare inherited metabolic disorder caused by defective glucose 6-phosphatase (G6Pase) activity. Although anemia, renal failure, and hepatic adenoma are the major clinical manifestations of GSDIa, there has been no report of refractory anemia in GSDIa patients on maintenance hemodialysis (HD) concomitant with multiple liver adenomas. Herein, we present a case of refractory anemia in a patient with GSDIa undergoing HD with multiple hepatic adenomas, successfully managed through aggressive treatment for renal anemia and intravenous iron therapy (IIT).

A 26-year-old man with GSDIa who had been on HD for a year suffered from refractory anemia. He had experienced hypoglycemia and hyperlactic acidemia repeatedly and unusual hypertriglyceridemia had been observed for a long time. In addition, multiple hepatic adenomas developed and his renal function gradually declined, eventually progressing to end-stage kidney disease, and HD was started. Despite 120 µg/week of darbepoetin alfa (DA), 200 mg/day of oral sodium ferrous citrate, and 600 mg/week of roxadustat, the anemia persisted and iron deficiency gradually progressed. We considered that renal anemia, blood loss by each HD session, and decreased intestinal iron absorption due to inappropriately increased hepcidin from hepatic adenomas were the main etiology of the anemia; hence, we changed oral sodium ferrous citrate to intravenous saccharated ferric oxide along with continuous aggressive treatment of renal anemia, and the anemia resolved quickly within three months.

We believe that refractory anemia was mainly induced by renal anemia and chronic iron deficiency due to blood loss during HD and inappropriately elevated hepcidin levels in hepatic adenomas. Aggressive treatment of renal anemia, along with IIT, may be a promising treatment option. Strict monitoring of iron overload is essential for safe treatment.

## Introduction

Glycogen storage disease type Ia (GSDIa), also known as von Gierke disease, is a rare inherited metabolic disorder caused by defective glucose 6-phosphatase (G6Pase) activity [1]. G6Pase is a key enzyme in carbohydrate metabolism. It hydrolyzes glucose 6-phosphate (G6P) to glucose and which is a pivotal step in both glycogenolysis and gluconeogenesis. Patients with GSDIa cannot produce glucose during periods of fasting, which results in excessive glycogen and fat accumulation, mainly in the liver and kidneys, along with laboratory abnormalities such as hyperlactic acidemia, hyperuricemia, and hyperlipidemia [2,3]. Although anemia, renal failure, and hepatic adenoma are the major clinical manifestations of GSDIa, there has been no report of refractory anemia in GSDIa patients on maintenance hemodialysis (HD) concomitant with multiple liver adenomas [4]. Herein, we present a case of refractory anemia in a patient with GSDIa undergoing HD with multiple hepatic adenomas, successfully managed through aggressive treatment for renal anemia and intravenous iron therapy (IIT).

## Case presentation

A 26-year-old man with GSDIa undergoing HD developed refractory anemia. The patient was diagnosed with GSDIa when he was an infant by genetic test. He had experienced hypoglycemia and hyperlacticacidemia repeatedly since childhood owing to poor treatment adherence to oral cornstarch supplementation, and unusual hypertriglyceridemia had been observed for a long time. He had enlarged liver, and multiple hepatic adenomas gradually developed during the long clinical course. His renal function gradually declined despite the conventional treatment by nephrologist, eventually progressing to end-stage kidney disease. He also had anemia refractory to a high dose of an erythropoiesis-stimulating agent (ESA), darbepoetin alfa (DA) 180 μg once every 2 weeks, and oral iron therapy (OIT) (sodium ferrous citrate 200 mg/day). Chronic inflammation was indicated by elevated C-reactive protein (CRP) levels (3-5 mg/dL), observed for a long duration. His medical history was unremarkable, except for GSDIa and its complications. His family history included his brother for GSDIa. He denied smoking or drinking. Except for the cornstarch supplementation, the patient’s medication adherence was relatively good.

Regarding renal replacement therapy (RRT), we recommended either simultaneous liver and kidney or isolated kidney transplantation; however, the patient declined both options. Although we considered peritoneal dialysis to be a more appropriate treatment than HD due to its additional property of mild glucose supplementation, he selected HD as an RTT.

On examination at the time of HD initiation, his height and bodyweight were 166.7 cm and 50.3 kg, respectively. His vital signs included a body temperature of 36.8℃, blood pressure of 149/95 mmHg, and pulse rate of 69 beats/min. A doll-like face and distended abdomen were observed. An enlarged liver was palpable. Laboratory data are in Table 1.

**Table 1:** Laboratory data of the patient WBC: white blood cell; RBC: red blood cell; Hb: hemoglobin; Ht: hematocrit; MCV: mean corpuscular volume; Reti: reticulocyte; PT-INR: prothrombin time international normalized ratio; APTT: activated partial thromboplastin time; T-Bil: total bilirubin; D-Bil: direct bilirubin; ALP: alkaline phosphatase; AST: aspartate aminotransferase; ALT: alanine aminotransferase; LDH: lactate dehydrogenase; GTP: glutamyl transpeptidase; ChE: cholinesterase; TG: triglyceride; cho: cholesterol; HDL: high-density lipoprotein; LDL: low-density lipoprotein; UA: uric acid; BUN: blood urea nitrogen; Cr: creatinine; NH3: ammonia; CRP: C-reactive protein; TIBC: total iron binding capacity; TSAT: transferrin saturation

Blood count	Results
WBC (/μL)	8,630
RBC (×10^4 ^/μL)	292
Hb (g/dL)	7.3
Ht (%)	24.6
MCV (fL)	84.2
Reti (%)	2.1
Platelets (×10^4 ^/μL)	31
Coagulation test	
PT-INR	0.93
APTT (s)	29.8
Biochemical and serological test	
Total protein (g/dL)	6.3
Albumin (g/dL)	2.6
T-Bil (mg/dL)	0.3
D-Bil (mg/dL)	0.1
ALP (IU/L)	166
AST (IU/L)	45
ALT (IU/L)	36
LDH (IU/L)	370
γ-GTP (IU/L)	255
ChE (IU/L)	281
Cr (mg/dL)	6.66
Na (mEq/L)	137.6
K (mEq/L)	6.9
Cl (mEq/L)	99.7
Ca (mg/dL)	6.7
P (mg/dL)	7.8
NH_3 _(μg/dL)	42
CRP (mg/dL)	4.92
Ferrum (µg/dL)	61
TIBC (µg/dL)	266
TSAT (%)	22.9
Folic acid (ng/mL)	10.8
Vitamin B12	10.8
Ferritin (ng/mL)	177.9

**Figure 1:**
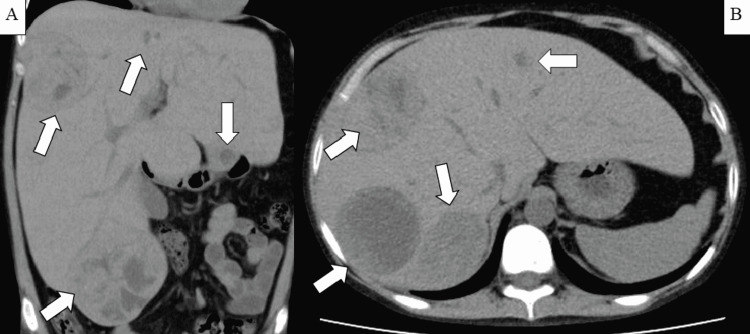
Chest-abdominal non-contrast computed tomography (A, coronal plane; B, transverse plane) The image shows enlarged liver with multiple adenomas.

Severe anemia, low reticulocyte count, hypertriglyceridemia, and elevated CRP levels were observed. Chest-abdominal non-contrast computed tomography (CT) revealed multiple hepatic adenomas without any infection focus, active bleeding, or malignancy (Figure 1).

**Figure 2:**
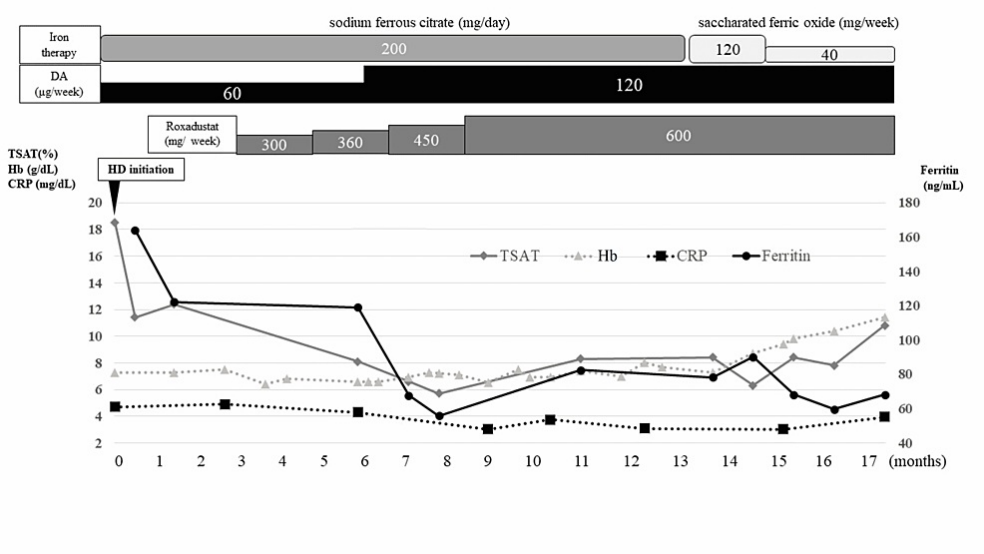
The clinical course of the anemia The anemia had been refractory to high dose of darbepoetin alfa (DA), sodium ferrous citrate, and roxadustat, which improved quickly following intravenous iron therapy initiation. TSAT: transferrin saturation; CRP: C-reactive protein; Hb: hemoglobin; HD: hemodialysis

Because there were no abnormal findings in physical examinations and CT other than known hepatic adenomas, hepatic adenomas were deemed the culprit inflammatory focus. Fecal occult blood tests over two consecutive days were negative. The clinical course of the anemia is presented in Figure 2.

We continued the administration of high doses of ESA and OIT. Because the patient had multiple hepatic adenomas associated with chronic inflammation, we did not change the oral OIT to IIT because of the possibility of iron overload and subsequent liver damage. The anemia did not improve despite the additional administration of hypoxia-inducible factor prolyl hydroxylase inhibitor (HIF-PH), roxadustat 300 mg/week. Finally, we used 120 µg/week of DA, 200 mg/day of sodium ferrous citrate, and 600 mg/week of roxadustat, along with adequate dialysis a year after HD initiation. However, the hemoglobin (Hb) levels remained at 6-8 g/dL. The patient’s iron deficiency was observed as decline in transferrin saturation and ferritin progressed gradually following HD initiation, and the CRP levels remained high (3-5 mg/dL). Repeated enhanced CT and fecal blood tests revealed no signs of bleeding, inflammatory focus, and malignancy other than known hepatic adenomas. Because a constant amount of residual blood on dialyzer had been observed despite the dialyzer change, we considered that this iron deficiency development was due to the blood loss in each HD session and a decrease in intestinal iron absorption due to hepcidin increase from multiple hepatic adenomas associated with chronic inflammation. Therefore, we changed the OIT to an IIT (saccharated ferric oxide) of 40 mg three times a week to supply iron directly, with strict monitoring for iron overload. Following IIT initiation, the anemia improved quickly. The Hb level reached 10 g/dL after three months of starting the ITT. No adverse events related to the ITT were observed.

Upon this clinical course, although we could not measure hepcidin, we considered that the patient’s refractory anemia was due to renal anemia and chronic iron deficiency resulting from blood loss during HD and decreased intestinal iron absorption due to hepcidin increase. This condition was successfully resolved by changing the OIT to IIT along with continuous aggressive treatment of renal anemia.

## Discussion

We describe a rare case of refractory anemia in a patient with GSD undergoing HD complicated by multiple hepatic adenomas, successfully treated with ESA, HIF-PH, and IIT. Although both the kidneys and the liver are frequently involved in GSD, the complications involving these organs that contribute to the development of severe anemia remain largely unknown, with no established treatment for similar conditions. Therefore, we believe our case could be useful in the treatment and management of similar cases.

Anemia is a major complication of GSDIa seen in over 80% of adult patients [5]. There are various factors in the pathogenesis of anemia in patients with GSDIa: malnutrition, chronic lactic acidosis, chronic kidney disease, bleeding tendency, impaired metabolic status, hepatic adenomas, and irritable bowel disease [4]. In our case, we consider that chronic kidney disease and chronic iron deficiency caused by blood loss in each HD and increased hepcidin levels due to multiple hepatic adenomas strongly contributed to the development of severe anemia.

Referring to renal involvement of GSDIa, nephrolithiasis due to hyperuricemia, increased urinary calcium excretion, decreased urinary citrate excretion, tubular dysfunction, and glomerular injury are the main pathogenesis of renal failure [4]. Aging, along with poor metabolic control, has been reported as a risk factor for progression of renal failure in previous studies. One study reported that 14 of 20 relatively older GSDI patients (aged 13-20 years) had renal function decline along with persistent proteinuria [6]. Of note, renal biopsy performed in three patients with a long history (> 10 years) of proteinuria revealed focal segmental glomerulosclerosis. Another study investigated the natural course of renal function in 39 patients with GSDI, disclosing early glomerular hyperfiltration followed by microalbuminuria, overt proteinuria, and a subsequent decline in glomerular filtration rate, similar to that observed in typical diabetic nephropathy [7]. In addition, less proteinuria was observed in the patients with optimal metabolic control. Furthermore, poor triglyceride control, along with aging, was reported to be associated with glomerular injury in another study [8]. Our patient was older and had a long history of poor metabolic control, which induced drastic progression of renal failure leading to end-stage kidney disease. Endogenous erythropoietin production was ultimately depleted [9]. Although we treated the patient with a high dose of ESA, HIF-PH, and OIT, anemia persisted. IIT successfully resolved anemia. Importantly, we believe that hepcidin elevation from hepatic adenoma was an additional pivotal factor in the development of severe anemia.

Regarding the hepatic manifestations of GSDIa, 100% of GSDIa patients in adulthood had hepatomegaly and 75% had hepatic adenomas, and both were shown in our patient [5]. Hepatic adenomas were identified mainly in the post-pubertal period. It is associated with an approximately 10% risk of malignant progression [10]. Metabolic status is reported to be a key factor associated with the development of hepatic adenomas along with other genetic factors [11,12], and fatty acid metabolism abnormality is hypothesized to be a key mechanism of adenoma formation [13]. In a retrospective study of 117 patients with GSDI, those with poor triglyceride control (five-year mean triglyceride concentration >500 mg/dL) had a higher rate of hepatic adenoma progression, while having better triglyceride control contributed to tumor regression [14]. Because fatty acid control had been extremely poor in our patient for a long time, he developed large multiple hepatic adenomas. A strong association between anemia and hepatic adenoma has been reported in previous studies. Wang et al. investigated the characterization and pathogenesis of anemia in 163 patients with GSDI and found that 75% of the patients with severe anemia (defined as the presence of Hb < 10 g/dL) had hepatic adenomas [15]. They concluded that imaging investigations of hepatic adenomas are essential in GSDI patients with progressive anemia. Hepcidin has been reported to be an inducing mediator in the pathogenesis of hepatic adenomas and anemia. Hepcidin is a peptide hormone comprising 25 amino acids. This peptide is essential for the maintenance of iron homeostasis. It inhibits both intestinal iron absorption and the efflux of iron from hepatic cells and macrophages via its iron-transporting receptor, ferroportin [16]. Hepcidin synthesis is mainly regulated by iron concentration and iron requirements in erythropoiesis and inflammation. Under normal conditions, iron deficiency leads to a decrease in hepcidin synthesis to increase iron delivery in the plasma [17]. In contrast, inappropriately high expression of hepcidin mRNA in hepatic adenomas in anemic GSDIa patients with iron deficiency has been reported [18]. In this study, hepcidin mRNA in non-affected liver tissue was appropriately suppressed, suggesting that the adenomas abnormally upregulate hepcidin expression independent of iron status, subsequently inducing impaired iron utility. Although we could not measure hepcidin, the clinical course suggests that inappropriately elevated hepcidin levels in multiple hepatic adenomas decrease intestinal iron absorption, resulting in chronic iron deficiency despite OIT, which then led to sustained severe anemia. Because there was no evidence of bleeding in enhanced CT and fecal occult blood tests, we considered that the emergence of iron deficiency was due to blood loss in every HD. This was successfully improved by IIT without no adverse events. Although we continued OIT instead of IIT at first over concern for risk of iron overload and subsequent liver damage in the background of multiple hepatic adenomas associated with chronic inflammation, IIT has recently been reported as a safe and effective therapy even in dialysis-dependent patients with apparent inflammation [19]. On the other hand, iron accumulation has been detected even in patients on HD within an acceptable range of serum ferritin levels by magnetic resonance imaging [20], and the ideal range of iron parameters in dialysis-dependent patients with apparent inflammation is yet to be determined [19]. From these studies, we consider it important to keep strict monitoring of iron parameters and liver function to prevent iron overload.

## Conclusions

We consider that poor long-term metabolic control contributed to the development of renal failure and hepatic adenomas in our case. We believe that refractory anemia was mainly induced by renal anemia and chronic iron deficiency due to blood loss during HD and inappropriately elevated hepcidin levels in hepatic adenomas. Aggressive treatment of renal anemia, along with intravenous IIT, may be a promising treatment option. Strict monitoring of iron overload is essential for safe treatment.
